# A joint finite mixture model for clustering genes from independent Gaussian and beta distributed data

**DOI:** 10.1186/1471-2105-10-165

**Published:** 2009-05-29

**Authors:** Xiaofeng Dai, Timo Erkkilä, Olli Yli-Harja, Harri Lähdesmäki

**Affiliations:** 1 Department of Signal Processing, Tampere University of Technology, Tampere, Finland

## Abstract

**Background:**

Cluster analysis has become a standard computational method for gene function discovery as well as for more general explanatory data analysis. A number of different approaches have been proposed for that purpose, out of which different mixture models provide a principled probabilistic framework. Cluster analysis is increasingly often supplemented with multiple data sources nowadays, and these heterogeneous information sources should be made as efficient use of as possible.

**Results:**

This paper presents a novel Beta-Gaussian mixture model (BGMM) for clustering genes based on Gaussian distributed and beta distributed data. The proposed BGMM can be viewed as a natural extension of the beta mixture model (BMM) and the Gaussian mixture model (GMM). The proposed BGMM method differs from other mixture model based methods in its integration of two different data types into a single and unified probabilistic modeling framework, which provides a more efficient use of multiple data sources than methods that analyze different data sources separately. Moreover, BGMM provides an exceedingly flexible modeling framework since many data sources can be modeled as Gaussian or beta distributed random variables, and it can also be extended to integrate data that have other parametric distributions as well, which adds even more flexibility to this model-based clustering framework. We developed three types of estimation algorithms for BGMM, the standard expectation maximization (EM) algorithm, an approximated EM and a hybrid EM, and propose to tackle the model selection problem by well-known model selection criteria, for which we test the Akaike information criterion (AIC), a modified AIC (AIC3), the Bayesian information criterion (BIC), and the integrated classification likelihood-BIC (ICL-BIC).

**Conclusion:**

Performance tests with simulated data show that combining two different data sources into a single mixture joint model greatly improves the clustering accuracy compared with either of its two extreme cases, GMM or BMM. Applications with real mouse gene expression data (modeled as Gaussian distribution) and protein-DNA binding probabilities (modeled as beta distribution) also demonstrate that BGMM can yield more biologically reasonable results compared with either of its two extreme cases. One of our applications has found three groups of genes that are likely to be involved in Myd88-dependent Toll-like receptor 3/4 (TLR-3/4) signaling cascades, which might be useful to better understand the TLR-3/4 signal transduction.

## Background

In the field of gene clustering, gene expression data has been widely used assuming that genes that have similar expression patterns should have similar cellular functions and are likely to be involved in the same cellular processes [1]. However, this assumption might be too simplistic considering the complexity of real biological systems. It has become more and more acknowledged that different data sources offer information from different perspectives, and their combinations might make the prediction more accurate. There are many types of biological data available besides gene expression data, such as protein-DNA binding data, protein-protein interaction data, evolutionary conservation data, gene ontology information, et cetera. However, different data types have different characteristics, and thus how to integrate multiple heterogeneous data types into a single framework and make the results more accurate has become one of the most challenging problems. In this study, we developed a clustering algorithm that can cluster genes based on beta distributed and Gaussian distributed data, which are represented by protein-DNA binding probabilities (predictions from a software [2]) and gene expression data, respectively, in a real case study. Other possible data sources that can be naturally modeled with beta distributions include e.g. correlations [3] and pair-wise and multiple sequence similarities [4], and other possible Gaussian distributed data sources include various other microarray-based measurements.

Many unsupervised methods have been developed and widely used in gene clustering. They can be roughly classified into three categories, which are heuristic, iterative relocation and model-based methods [5]. The first two approaches suffer from solving some basic practical issues such as 'how to define the number of clusters' and 'how to handle outliers', which can be easily handled by model-based methods. For the first issue, the problem can be recasted as the model selection problem; and for the second question, the outliers can be handled by adding one or more components which represent a different distribution for them [3,5]. Moreover, model-based clustering methods outweigh approaches within the other two categories in their statistical nature [5]. So in this study, we choose model-based clustering as the framework for the unsupervised data fusion.

Expectation maximization (EM) algorithm is often used to solve the problem of maximum likelihood estimation with incomplete data, and thus is commonly adopted in model-based clustering. Although EM algorithm for Gaussian distribution is well-known, less information is available about that for other distributions, not mentioning combinations of different distributions. In this study, beta distributed data and Gaussian distributed data are integrated into one combined mixture model. We have developed three types of EM algorithms, the standard EM (EM_*s*_), an approximated EM (EM_*a*_) and a hybrid EM (EM_*h*_) algorithm for BGMM, whose comparisons were done using simulated data. EM_*h *_was used for BGMM in the simulations and real case studies. Performance tests with BGMM and its component models (BMM, GMM) were done both with simulated and real data, and the results show that our joint mixture model can yield more accurate results. These results also demonstrate the idea that the more data that are integrated the more comprehensive the result will be.

Two commonly used model selection criteria are likelihood-based methods and approximation-based methods, of which approximation-based methods are widely preferred due to their simplicity and less computational cost [6]. These methods include penalized likelihood, closed-form approximations to the Bayesian solution, and Monte Carlo sampling of the Bayesian solution, among which the first two methods are most prevalent. Four well-known model selection criteria, Bayesian information criterion (BIC), integrated classification likelihood-BIC (ICL-BIC, we call it ICL for simplicity in this paper), Akaike information criterion (AIC), and modified AIC (AIC3) were tested for BGMM and its two extreme models in this study. ICL is reported to work well for BMM [3], and AIC as well as BIC are commonly used as the criterion in GMM [3,7]. Our simulation results in this study suggests that AIC or ICL is preferred in BGMM depending on which EM algorithm is employed.

The following sections are organized as 'Methods', 'Results and Discussion', and 'Conclusions'. In section 'Methods', we introduced BGMM together with all its three types of EM algorithms, and described the formulation of the four tested model selection criteria. In section 'Results and Discussion', we first compared the three types of EM algorithms in BGMM (where EM_*h *_is chosen to be used in the simulations and real case studies), and then compared the performance of BGMM with BMM and GMM. In section 'Conclusions', we first summarized the main work of this study, discussed the possible extension and limitations of the current work, and in the end briefly mentioned the related future work.

## Methods

In this section, BGMM and all its three types of EM algorithms are first introduced, and then the approximation based model selection criteria which are compared in this study are described in detail.

### Mixture model based clustering

In model-based clustering methods, each observation **x**_*j*_, where *j *= 1,..., *n *and *n *is the number of genes, is drawn from a finite mixture distribution with the prior probability *π*_*i*_, component-specific distribution  and its parameters *θ*_*i*_. The formula is given as [8]

(1)

where *θ *= {(*π*_*i*_, *θ*_*i*_}: *i *= 1,..., *g*} is used to denote all the unknown parameters, with the restriction that 0 <*π*_*i *_≤ 1 for any *i *and . Note that *g *is the number of components in this model. In the following texts, we ignore the superscript (*g*) from  for simplicity.

### BGMM

In BGMM, we define *θ *= [*π*, *θ*_1_, *θ*_2_]^*T*^, *π *= [*π*_1_,..., *π*_*g*_]^*T*^,  and ], where *p*_1 _and *p*_2 _each represents the dimension of the observations in BMM and GMM, respectively. We also denotes *Y *and *Z *as the observations of beta distributed and Gaussian distributed data, respectively, function *f *of **y **and *f *of **z **as the density function of beta and Gaussian distribution, respectively, and **x **= [**y**^*T*^, **z**^*T*^]^*T*^. *Y *and *Z *can be used to denote different data sources in different contexts, which for example denote TF binding probabilities and gene expression data in our bioinformatics application.

BGMM is built from BMM and GMM with the assumption that, for each component *i*, the beta distributed and Gaussian distributed data are independent. In the BMM part, each component is assumed to be the product of *p*1 independent beta distributions, whose probability density function is defined as

(2)

where  and . Likewise, each component is assumed to follow a Gaussian distribution in the GMM part, whose probability density function of each component for each gene is defined as

(3)

where , ,  and . We assume the commonly used Gaussian model where the covariance matrix is a diagonal matrix. This approximation is useful especially for high-dimensional data since it significantly reduces the number of parameters that need to be estimated from data. It is worth noting that the above mixture model construction implicitly assumes that the two data sources share the same clustering structure, which is a reasonable assumption for the general problem of clustering gene expression and TF binding data (see, e.g., [9]). However, this assumption does not necessarily hold in all other clustering problems, in which case our method is not applicable (see the Section 'Conclusions' for further discussion).

EM algorithm is applied to estimate the parameters *θ *iteratively. We have developed three types of EM algorithms for BGMM, the standard EM (EM_*s*_), an approximated EM (EM_*a*_) and a hybrid EM (EM_*h*_), which are described in detail in the following sections.

### EM algorithms

#### The standard EM algorithm

In the standard EM algorithm, the data log-likelihood (natural logarithm is referred to throughout this paper) can be written as

(4)

given *X *= {**x**_*j*_: *j *= 1,..., *n*}, whose direct maximization, however, is difficult.

In order to make the maximization of Equation 4 tractable, the problem is casted in the framework of incomplete data. Since we assume that the beta distributed and Gaussian distributed data are independent, *L*_*c *_can be factored as

(5)

If we define *c*_*j *_∈ {1,..., *g*} as the clustering membership of **x**_*j*_, then the complete data log-likelihood can be written as

(6)

where *χ*(*c*_*j *_= *i*) is the indicator function of whether **x**_*j *_is from the *i*^th ^component or not.

In the EM algorithm, E step computes the expectation of the complete data log-likelihood

(7)

where *θ*^(*m*) ^represents the parameters estimated in the *m*^th ^iteration (derivation of *Q *is referenced from [8]). By computing the expectation, Equation 7 becomes

(8)

where

(9)

according to Bayes' rule. Note that  is the estimated posterior probability of **x**_*j *_coming from component *i *at iteration *m*, and we can assign each **x**_*j *_to its component based on . Equations 7 and 8 show that our assumption of the beta distributed and Gaussian distributed data being independent carries over to the expected log-likelihood as well.

In the EM algorithm of BGMM, *α*_*iu*_'s and *β*_*iu*_'s, which are the parameters of the BMM part, are estimated using Newton-Raphson method. Let *θ*_1*i *_= (*α*_*i*_, *β*_*i*_), then the parameters are updated by

(10)

with the constraint *θ*_1*i *_≥ **1**, where  is the Hessian matrix evaluated at  and  is the Lagrangian function of  (derivations shown in Appendix). The parameters of the GMM part, *μ*_*iv*_'s and , in BGMM can be estimated by the standard EM algorithm of GMM with diagonal covariance matrix, which works by iterating over (derivations are referenced from [8])

(11)

(12)

and *π*'s are updated by

(13)

where  is calculated from Equation 9 (derivation shown in Appendix). Note that {*u *= 1,..., *p*_1_} and {*v *= 1,..., *p*_2_}.

From the above equations, it is easy to see that the standard EM for BGMM will reduce to the standard EM for BMM when *p*_2 _goes to 0 and shrink to the standard EM for GMM when *p*_1 _= 0.

#### Approximated and hybrid EM algorithms

We also developed an approximated EM algorithm for BGMM, whose main difference compared with the standard one is that it maximizes Equation 6 instead of Equation 7.

In E step, *τ*_*ji*_'s are first calculated with the current parameters, according to which **x**_*j*_'s are clustered to their corresponding clusters using *c*_*j *_= *i*_0 _where *i*_0 _= arg max_*i *_*τ*_*ji*_. Then in M step, the new parameters are estimated so as to maximize Equation 6 (in maximum likelihood sense) given the hard clusters obtained in E step. Given that the beta and Gaussian distributed data are assumed to be independent, ML parameter estimates for beta and Gaussian parts can be computed separately, which corresponds to the basic ML estimation using standard techniques. In the approximated EM, the new  and  are estimated with a numerical optimization method, 'betafit', which is implemented in matlab, and the new  and  are calculated by

(14)

(15)

respectively, where  is composed of all the genes in cluster *i *estimated from E step,  refers to the  of cluster *i*, and . Update of *π*_*i*_'s and calculation of *τ*_*ji*_'s remain the same with the standard EM algorithm.

In the end, we developed one type of hybrid EM algorithm, whose *α*_*iu*_'s and *β*_*iu*_'s are maximized by the approximated EM, *μ*_*iv*_'s,  and *π*_*i*_'s are updated by the standard EM.

The approximate EM for clustering is analogous to the Viterbi training for hidden Markov models (HMM). Viterbi training has been proposed as an alternative to the standard EM in the cases where the standard EM becomes computationally too expensive. Although there are no convergence guarantees in general, the Viterbi training has been found useful due to its efficiency and, in particular, when one seeks to decode the state (path) via Viterbi algorithm. The same considerations apply for the clustering problem as well, where the approximate EM optimizes the hard clustering and parameters iteratively. Moreover, because parameter estimates remain fixed for a given hard clustering, the optimization is a discrete process and, therefore, convergence is achieved exactly. The hybrid method shares (approximately) the benefits from both the standard EM and the approximate EM.

In order to avoid the possible local maxima, we run the algorithm (all the three types of EM algorithms) multiple times with different initial values. The parameters *α*_*iu*_'s and *β*_*iu*_'s for each dimension of the beta distribution *u *(*u *∈ {1,..., *p*_1_}) are initialized by method-of-moments so that their means are randomly distributed within the range of *y*_1*u*_,..., *y*_*nu *_and variances are equal for all clusters (*g*), *μ*_*iv*_'s and  are obtained from the randomly initialized fuzzy c-means clustering results, and *π*_*i*_'s are initialized with the uniform probability 1/*g*.

In this study, for each data set, we run each EM algorithm 100 times with different initial values, and for all the tested models, we set the convergence threshold (where the absolute difference of *Q *is used to monitor the convergence) and maximum number of iterations to 0.0001 and 100 respectively. All the simulations have reached their convergence according to the statistics stored during the simulations.

### Model selection

Four well-known approximation-based model selection criteria, BIC [10,11], ICL [3], AIC [7,12], and AIC3 [7,13] are compared in BGMM and its extreme models, according to which the best-performing criterion for each model is chosen. Calculations for the above criteria are defined as

(16)

(17)

(18)

(19)

where *d *is the number of free parameters, and *M *(in equations 18 and 19) is the total amount of the data (, *M*_*w *_is the size of data set *w *and *W *is the number of input data sets). Note that  is the estimated entropy of the fuzzy classification matrix *C*_*ji *_= (*τ*_*ji*_) [3].

The number of free parameters *d *are distinct in different models. In BMM, we have *p*_1_*g *free *α*_*iu*_'s, *p*_1_*g *free *β*_*iu*_'s, and *g *- 1 free *π*_*i*_'s (), so *d*_*B *_= 2*p*_1_*g *+ *g *- 1. In GMM, as we have *p*_2 _free *σ*_*v*_'s, *p*_2_*g *free *μ*_*iv*_'s, and also *g *- 1 free *π*_*i*_'s, thus *d*_*G *_= *p*_2 _+ *p*_2_*g *+ *g *- 1. In the joint model, the number of free parameters is the summation of those in its extreme models minus one set of free *π*_*i*_'s, therefore we have *d*_*BG *_= 2*p*_1_*g *+ *p*_2 _+ *p*_2_*g *+ *g *- 1.

## Results and discussion

In this section, we first compared the performance of BGMM with different EM algorithms by artificial data, according to which one EM was chosen for later simulations. Then we tested the integration idea (the more data sources that are integrated the more reasonable the results turn out to be) by comparing BGMM with its two extreme cases.

### Performance test of BGMM with artificial data

To evaluate the overall performance of a clustering method, we developed one scoring system to evaluate the clustering accuracy when dealing with artificial data. It searches the best matching between the cluster labels of the results (selected by the model selection criterion) and the ground truth clustering among all of their possible associating ways. The score for the best match is denoted as 'E score', and is defined as

(20)

In this scoring system, *T*_*j *_denotes the ground truth clustering membership of data *j*; *R *stands for all possible associating ways between the estimated and the true clusters, where *r*_*i *_is the label of data belonging to component *i *predicted by the clustering algorithm, and *r *is chosen from labels 1, 2,...,max{, g} ( and *g *are the largest labels in the estimated and ground truth clustering respectively). Also note that *e *represents the individual score of each gene, *E *is the average score of all the genes for each repetition, 'E score' of each repetition is the one corresponding to the optimal *Q*, and the final 'E score' of each data set is the median of the 10 'E score's. It is worth noticing that the estimated and assumed number of clusters,  and *g*, vary with the model selection criteria, and thus cause different 'E scores', rendering this scoring system not only records the accuracy of the results but also reflects the influence of model selection criterion.

#### Performance test of different EM algorithms in BGMM

**Figure 1:**
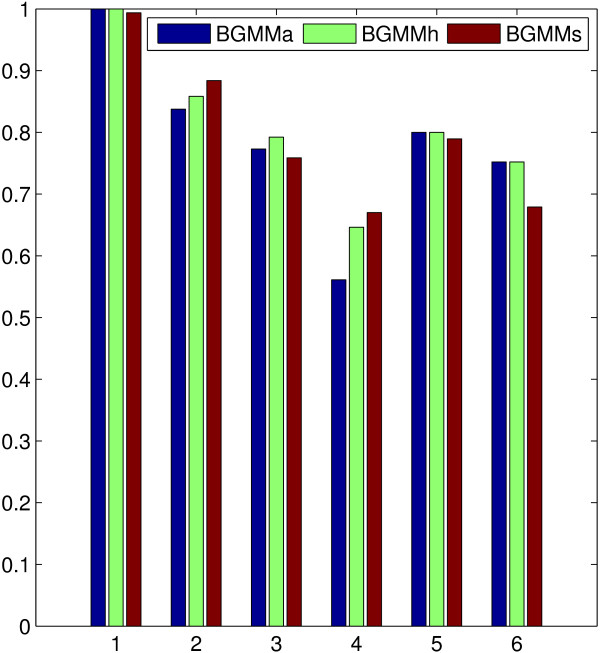
**Comparison of the E score among BGMM_*a*_, BGMM_*h *_and BGMM_*s*_**. x-axis corresponds to the different combinations of the tested scenarios: 1:gB+gG, 2:bB+gG, 3:gB+bG_*m*_, 4:bB+bG_*m*_, 5:gB+bG_*v*_, 6:bB+bG_*v*_.

We first compared the performance of EM_*s*_, EM_*a *_and EM_*h *_in BGMM. For simplicity, we denote BGMM that employs EM_*s*_, EM_*a *_or EM_*h *_as BGMM_*s*_, BGMM_*a *_or BGMM_*h*_, correspondingly. The artificial data set for the performance test was designed according to our model, whose parameters are listed in Table 1. The data set was divided into high quality (good) and low quality (bad) data, namely 'gB' (good, Beta distribution), 'bB' (bad, Beta distribution), 'gG' (good, Gaussian distribution) and 'bG' (bad, Gaussian distribution) respectively. We also designed two kinds of 'bG's, 'bG_*m*_' and 'bG_*v*_', which were hard to be clustered compared to 'gG' with respect to close means and large variances, respectively. The data set was designed to have three underlying clusters, 100 genes (*n *= 100) and four features (*p*_1 _= *p*_2 _= 4). The simulation was repeated 10 times with randomly generated data sets, and the comparison results of the clustering accuracy were depicted in Figure 1.

**Table 1:** Dataset designed for comparing different EM algorithms in BGMM

		cluster 1				cluster 2				cluster 3			
gB	alpha	15	20	25	20	20	25	15	5	1	20	1	30
	beta	20	15	20	25	20	25	15	5	20	1	30	1
	
bB	alpha	15	10	25	20	10	5	15	12	30	25	30	35
	beta	10	15	20	25	5	10	12	15	25	30	35	30

gG	mean	9	-9	11	-11	10	-10	12	-12	11	-11	13	-13
	variance	0.1	0.2	0.15	0.25	0.1	0.2	0.15	0.25	0.1	0.2	0.15	0.25
	
bG_*m*_	mean	9.1	-9.1	11.1	-11.1	9.2	-9.2	11.2	-11.2	9.3	-9.3	11.3	-11.3
	variance	0.1	0.2	0.15	0.25	0.1	0.2	0.15	0.25	0.1	0.2	0.15	0.25
	
bG_*v*_	mean	9	-9	11	-11	10	-10	12	-12	11	-11	13	-13
	variance	1	2	1.5	2.5	1	2	1.5	2.5	1	2	1.5	2.5

'gB', 'bB' and 'gG' stand for good, bad beta distributed data and good Gaussian distributed data re-spectively; 'bG_*m*_' and 'bG_*v*_' represent bad Gaussian distributed data which are hard to be clustered with respect to close means and large variances respectively.

In order to choose the best model selection criterion (with the highest E score) for each type of BGMM, we summed up the number of hits of the correct number of clusters for each tested case. The summation results for BIC, ICL, AIC and AIC3 are 24, 26, 17 and 19, respectively, in BGMM_*s*_, 23, 22, 29 and 23, respectively, in BGMM_*a*_, and 16, 16, 30 and 21, respectively, in BGMM_*h*_. Therefore, ICL is upheld by BGMM_*s*_, and AIC is embraced by both BGMM_*a *_and BGMM_*h *_in this simulation.

We evaluated the clustering accuracy of different types of BGMM with each best model selection criterion. Simulation results show that, although different algorithms perform slightly different for different cases (small performance differences can also depend on how well different algorithm converge to global maximum), the overall prediction accuracy of the three methods are similar as shown in Figure 1. We also compared the running time of the three methods under the same background framework, where no significant difference among them was detected.

**Figure 2:**
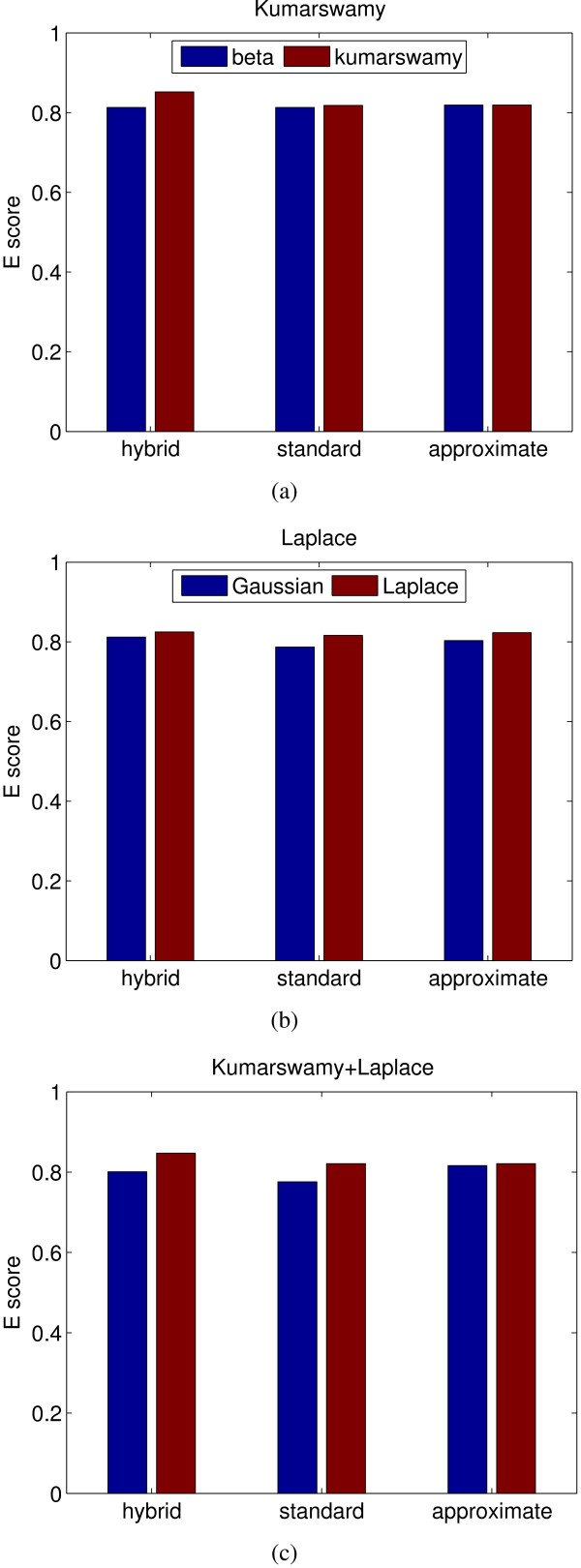
**Robustness test of BGMM_*a*_, BGMM_*h *_and BGMM_*s*_**. (a) Beta distribution replaced with Kumaraswamy distribution. (b) Gaussian distribution replaced with Laplace distribution. (c) both (a) and (b). x-axis corresponds to the different EM algorithms.

One important application of the proposed algorithm is to cluster genes based on protein-DNA binding probabilities and gene expression data, which are assumed to be of beta and Gaussian distributions in BGMM. This parametric assumption is supported by our good clustering results and additional distributional assessments. In some cases, however, our parametric assumptions might be violated due to various reasons, especially for expression data. For example, different platforms used to measure transcriptome might affect the distribution of expression data. Although this problem can be solved by extending the current algorithm to other parametric distributions quite easily, it is important to know how sensitive BGMM is to the violation of the parametric assumptions and how robust the algorithm is in dealing with noisy variables. To address this, we run three additional simulations with the three EM algorithms, where gene expression and protein-DNA binding data are simulated from Laplace and Kumaraswamy distributions, respectively. Laplace and Kumaraswamy distributions are used to replace Gaussian and beta distributions separately in simulation 1 and 2, and both distributions are replaced in simulation 3. Note that Laplace and Kumaraswamy distributions have the same support as Gaussian and beta distributions, respectively. Means and variances used in Laplace distribution are the same with those of Gaussian distribution ('gG' in Table 2), and *α *'s and *β *'s used in Kumaraswamy distribution are also the ones used in beta distribution ('gB' in Table 1). As shown in Figure 2, all three EM algorithms work similarly, and are not excessively sensitive to the parametric assumptions used in this study.

**Table 2:** Redesigned part of the data set used for comparing BGMM with BMM and GMM

		cluster 1				cluster 2				cluster 3			
gG	mean	9	-9.5	11	-11	9.5	-10	11.5	-11.5	10	-10.5	12	-12
	variance	0.5	0.6	0.7	0.8	0.5	0.6	0.7	0.8	0.5	0.6	0.7	0.8
	
bG_*m*_	mean	9.1	-9.1	11.1	-11.1	9.2	-9.2	11.2	-11.2	9.3	-9.3	11.3	-11.3
	variance	0.5	0.6	0.7	0.8	0.5	0.6	0.7	0.8	0.5	0.6	0.7	0.8
	
bG_*v*_	mean	9	-9.5	11	-11	9.5	-10	11.5	-11.5	10	-10.5	12	-12
	variance	1.5	2	2.5	3	1.5	2	2.5	3	1.5	2	2.5	3

Parameters of the beta distributed data and the symbols are the same with what has been shown in Table 1.

Based on the above test, the three EM algorithms perform equally well. Therefore, we simply used BGMM_*h *_for the performance tests and referred to it as 'BGMM' for simplicity in the following text.

#### Performance test of BGMM with its component models

Simulations shown in this section were dedicated to test how well BGMM could integrate different data sources. We compared the performance of BGMM (hybrid version, which is composed of approximated EM for the beta component and the standard EM for the Gaussian component) with its two extreme models, BMM with EM_*a *_(referred to as BMM) and GMM with EM_*s *_(referred to as GMM), for this purpose. A slightly different data set was used, where the Gaussian distributed data was designed to be less distinguishable than what has been shown in the previous section. The parameters of the redesigned data are shown in Table 2, where all the rest information including the dimensions of the data (*n *= 100 and *p *= 4) and the repetitions (10 times) remain the same.

We used the same method as what we did in the previous section to select the best criterion for BGMM, BMM and GMM. Ordered by BIC, ICL, AIC and AIC3, the summations of the hits are 0, 0, 23, 14, respectively, in BGMM, 3, 0, 30, 13, respectively, in BMM, and 0, 0, 10, 4, respectively, in GMM, according to which AIC was chosen as the best criterion for all the three tested models in this simulation.

**Figure 3:**
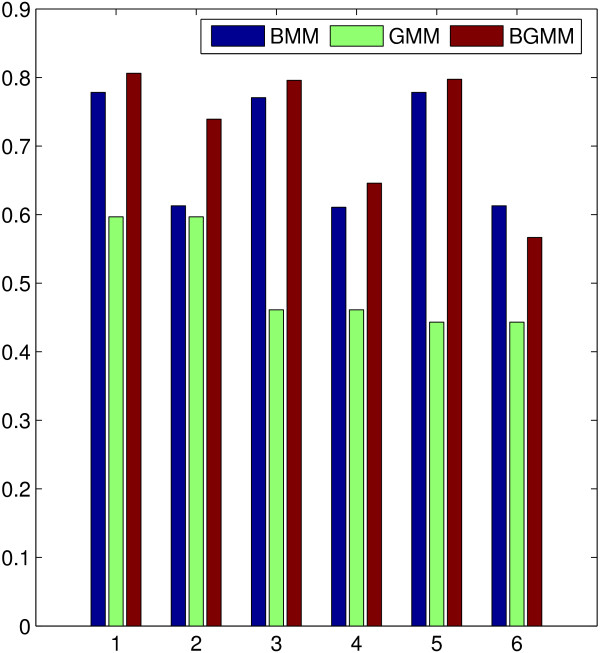
**Performance comparison of BGMM with BMM and GMM**. x-axis corresponds to the different combinations of the tested scenarios: 1:gB+gG, 2:bB+gG, 3:gB+bG_*m*_, 4:bB+bG_*m*_, 5:gB+bG_*v*_, 6:bB+bG_*v*_.

The comparison results of BGMM with its extreme models are shown in Figure 3. For expression data whose variances are not too large, the joint model can improve the clustering accuracy regardless of the quality of the data compared with either of its extreme models (E scores for cases 'gB+gG', 'bB+gG', 'gB+bG_*m*_' and 'bB+bG_*m*_' in BGMM are higher than those in BMM or GMM). However, when Gaussian distributed data has too much overlap among the clusters, BGMM does not necessarily show its superiority (compared to both BMM and GMM) when the variances are too large as shown in the case of bG_*v*_. It is indicated that BGMM is sensitive to the variances of Gaussian distributed data since bG_*v *_is designed to have similar noise level as that of bG_*m*_. These results demonstrate that the EM algorithm of BGMM has the power of reinforcing each extreme model with information from the other one, but does not necessarily outweigh both of them if the Gaussian distributed data contains too much noise with respect to large variances ('gB+bG_*v*_', 'bB+bG_*v*_').

### Performance test of BGMM with real data

We applied our methods to mouse protein-DNA binding data and gene expression data. The binding data is modeled as beta distribution, which are the binding probabilities output from a method called 'ProbTF' [2]. ProbTF uses genome sequences and transcription factor sequence specificities to compute the protein-DNA binding probabilities. This method answers the question of whether the whole gene promoter has one or more binding sites for a TF. Since it processes each promoter as a whole, the computational predictions provide insights into the functional role of a TF in the regulatory program of a target gene. The rationale for this is the fact that the higher binding probability anywhere on the promoter (not just in a particular location) implies higher probability of a regulatory relationship. Further, the method is able to make use of practically any genome-level information, such as evolutionary conservation, nucleosome positioning, ChIP-chip, and other prior knowledge (for more details, see [2]). The protein-DNA binding data contains the probabilities of 266 TFs binding to 20397 genes, calculated with mouse-specific position weight matrices from the TRANSFAC database (the web server is available at [2]). The gene expression data is modeled as Gaussian distribution, which is composed of 1960 genes measured from 95 conditions [14]. There are 1775 genes measured in both data sets. We removed the genes whose gene expression profiles have low absolute values (less than 10th percentile) with matlab function 'genelowvalfilter', and then choose genes that have annotations available for sure with the functional classification tool of DAVID database (the web server is available at [15]). In the end, we obtained 673 genes for the following studies.

**Figure 4:**
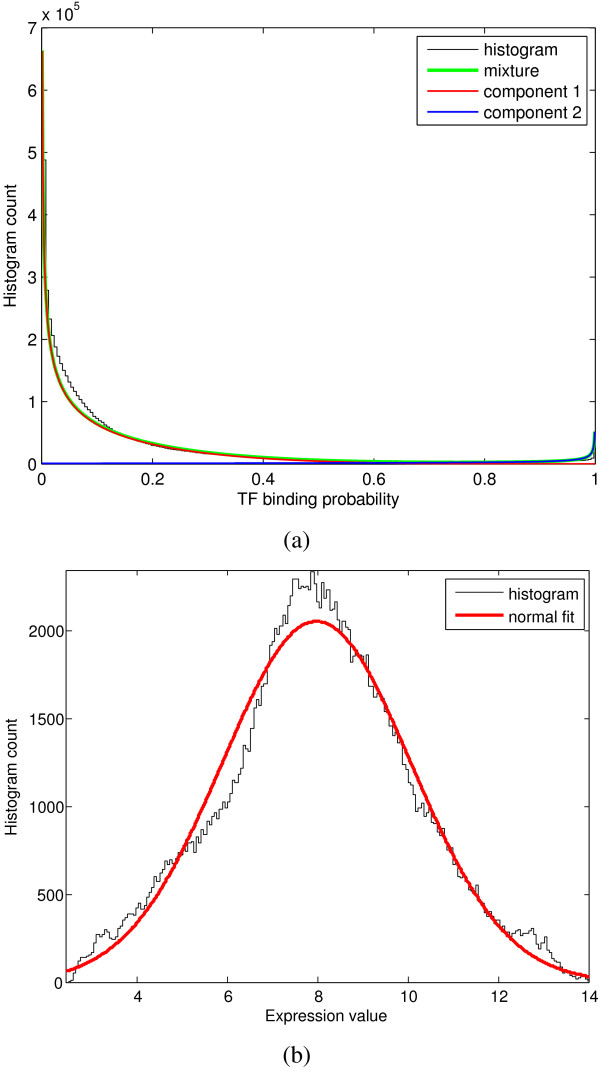
**Assessment of parametric assumptions**. (a) Genome-wide protein-DNA binding data fitted with two-component beta mixture model which has been estimated with the proposed EM algorithm. (b) Genome-wide gene expression data fitted with a Gaussian distribution. In both cases the standard histogram is shown as a reference distribution.

To see how well our data satisfy the parametric assumptions, we did the following test. For protein-DNA binding data, we grouped all the binding probabilities (20397 × 266) into two beta-distributed clusters (using the proposed method) and drew their PDFs, each representing the binding and unbinding cases, respectively. Figure 4(a) shows that the genome-wide binding data can remarkably well be approximated with two beta-distributed components. Similarly as shown in Figure 4(b), expression data can be fitted into a Gaussian distribution. This agrees with previous studies where gene expression data from a microarray platform is commonly assumed to be normally distributed. Although the above preliminary test does not correspond to our clustering method exactly, it demonstrates that our parametric assumptions are indeed reasonably good. The BGMM clustering method effectively increases the number of clusters to which the data is split and further improves the fit to the data.

The binding data corresponding to two sets of TFs were chosen to cluster the genes together with its corresponding expression data by BGMM, BMM and GMM. The clustering results were then compared and evaluated by Gene Ontology (GO). The first set of TFs was randomly chosen with respect to their biological significance (called 'Set_*rand*_'), while the second set was carefully selected by our model (named 'Set_*real*_'). There are three subsets of 'Set_*rand*_', each of which was chosen based on certain criterion. We arbitrarily choose three thresholds to be compared with the median of the binding probabilities of a certain TF, and TFs that exceed this threshold are used for clustering (using thresholds is just a way to define different levels of binding specificity to the choice of TFs). The thresholds for 'Set_*rand*1_' to 'Set_*rand*3_' are 0.3, 0.4 and 0.5, respectively, and the number of TFs selected are 11, 3 and 1, correspondingly. 'Set_*real*_' was selected by BMM. We first clustered the genes based on two sets of TFs by BMM, which were Bach1 and Bach2 combined with MafK, respectively. This is because that the two Bach proteins are both reported to interact with MafK protein. Then we compared the genes whose cluster has the lowest enrichment score from each clustering result, and the common set which contains 44 genes was chosen. We further clustered all the 266 TFs based on the 44 genes by BMM, and focused on the cluster that contains Bach1, Bach2 and MafK. This cluster turns out to be composed of all the TFs that belong to the families Fos, Jun, Maf and NF-E2 among our tested TFs, which are all AP-1(-like) components of the Leucine zipper factors class. There are 19 TFs (AP1, Fos, Fosb, Fosl1, Fosl2, Jun, Junb, Jund1, Maf, Mafb, Maff, Mafg, Mafk, Bach1, Bach2, Nfe2, Nfe2l1, Nfe2l2 and Nfe2l3) in this cluster, all of which were chosen to form 'Set_*real*_'.

Maf family proteins (contains Maf, Mafb, Maff, Mafg, Mafk) heterodizes with CNC-related bZip factors which include NF-E2 family proteins (includes Bach1, Bach2, Nfe2, Nfe2l1, Nfe2l2 and Nfe2l3) [16,17]; while Fos family (contains Fos, Fosb, Fosl1, Fosl2) form hetero (Fos-Jun; the heterodimer is also called AP1) or homo (Jun-Jun) dimers with Jun family (includes Jun, Junb, Jund1) proteins [18]. These dimers bind to DNA at certain motif that contains AP-1 binding sites [16-18]. The result that our BMM can cluster TFs which have similar binding profiles into one single cluster demonstrates the applicability of BMM, one extreme case of BGMM.

GO was employed in this study to validate the clustering results. In order to find the most significant annotated terms by looking at the probabilities that the terms are counted by chance, we used the hypergeometric probability distribution to calculate the p-values of gene enrichment score (called 'p-values' for simplicity) for each cluster by each model with each model selection criterion (Bioinformatics Toolbox 3.1 in Matlab). We compared the means and medians of those p-values across all the groups clustered by each model, whose results are shown in Table 3. It is worth mentioning that the clustering result is obtained by running the algorithm 100 times and taking the one whose expected complete data log-likelihood is the maximum, and each p-value shown in Table 3 is the mean or median of the p-values of all the ontology groups (from Gene Ontology) corresponding to the best clustering result (selected by its corresponding model selection criterion). From this table, it is clear that, no matter whether the TFs were randomly selected or not, both means and medians of the p-values of BGMM are lower than those of either BMM or GMM, regardless of which aspect ('All', 'F','C','P') was considered and which model selection criterion was used. These results indicate that our BGMM can cluster the genes in a more reasonable way with respect to their biological functions, localizations and processes involved. It is also seen from Table 3 that, there are two cases where the four model selection criteria have different prediction results, one is in BMM of the case Set_*rand*2 _where the results chosen by AIC yields the smallest p-values, and the other is in GMM of the case Set_*real *_where AIC selects the best model in terms of the smallest p-value, both of which accord well with our simulation results. Moreover, the choice of TFs whose binding probabilities are used in clustering does obviously affect the results and, therefore, TFs should be carefully chosen based on biological knowledge of a specific problem. In this study, although binding data of randomly (i.e., without prior biological knowledge) chosen TFs (Set_*rand*_) also give lower p-values, the obtained clusters might not provide best insight into our biological problem. We therefore carefully studied the results obtained from Set_*real*_, which are discussed below.

**Table 3:** Comparison results of BGMM, BMM and GMM in applications to real data

			All		F		C		P	
Dataset	Model	Criterion	Mean	Median	Mean	Median	Mean	Median	Mean	Median

	BMM	4	0.2094	0.2246	0.3410	0.3453	0.3512	0.3552	0.3417	0.3404
Set_*rand*1_	GMM	4	0.1958	0.1658	0.26719	0.3091	0.2925	0.3408	0.2747	0.3398
	BGMM	4	0.1568	0.1047	0.2347	0.1826	0.2663	0.2536	0.2451	0.2287

		BIC/AIC3	0.2071	0.1863	0.3261	0.3490	0.3408	0.3505	0.3331	0.3585
	BMM	ICL	0.2013	0.2013	0.3080	0.3080	0.3631	0.3631	0.3594	0.3594
Set_*rand*2_		AIC	0.1634	0.1505	0.2699	0.2499	0.2890	0.2772	0.2727	0.2427
	GMM	4	0.1958	0.1658	0.2672	0.3091	0.2925	0.3408	0.2747	0.3398
	BGMM	4	0.1436	0.0954	0.2198	0.2199	0.2453	0.2526	0.2311	0.2409

	BMM	4	0.2204	0.2204	0.3748	0.3748	0.3799	0.3799	0.3769	0.3769
Set_*rand*3_	GMM	4	0.1958	0.1658	0.2672	0.3091	0.2925	0.3408	0.2747	0.3398
	BGMM	4	0.1466	0.1155	0.2623	0.2551	0.2838	0.3036	0.2714	0.2811

	BMM	4	0.2407	0.2414	0.3228	0.3055	0.3575	0.3695	0.3300	0.3442
Set_*real*_	GMM	BIC/ICL	0.1973	0.1957	0.2799	0.2999	0.3040	0.3486	0.2883	0.3214
		AIC/AIC3	0.1882	0.1708	0.2747	0.2917	0.3010	0.3325	0.2813	0.3103
	BGMM	4	0.0987	0.0610	0.2455	0.2170	0.2894	0.2999	0.2558	0.2658

Statistics shown in this table are the group average of the p-values of gene enrichment score (each p-value is the average of the p-values of all the ontology groups corresponding to the best clustering result selected by its corresponding model selection criterion). TFs of 'Set_*rand*1_' to 'Set_*rand*3' _were randomly chosen according to certain thresholds (details shown in the text), while TFs of 'Set_*real*_' were carefully selected by BMM.

Note: 'F', 'C' and 'P' stand for the three aspects of gene ontology; 'All' means all aspects are included. '4' represents that all the four criteria indicate the same clustering result. Statistics shown here are all rounded to 4 decimals.

**Figure 5:**
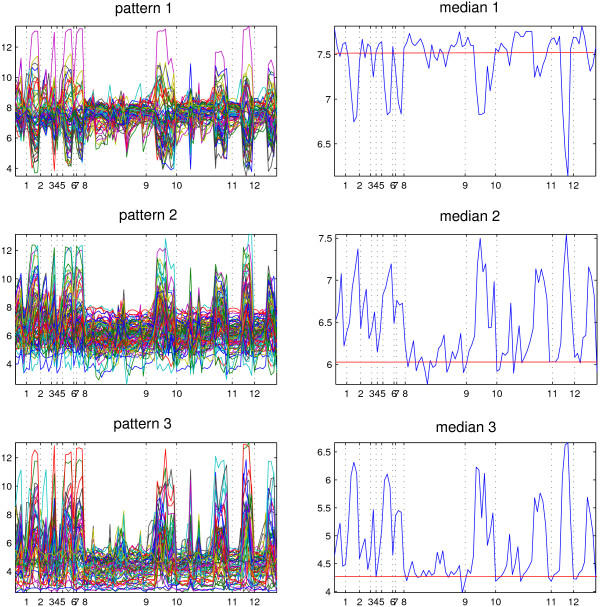
**Expression patterns of gene groups 'clu1' to 'clu3' clustered by BGMM**. x-axis corresponds to different treatments, which have been divided into different regions by 12 points; y-axis stands for the expression level; red horizontal bar symbolizes the average expression level of the group of genes it represents without external stimuli.

There are eight clusters obtained from Set_*real *_by BGMM, among which three groups have p-values below 0.05 if all the aspects were taken into account (without multiple testing correction). The three clusters were named 'clu1' to 'clu3' and ordered from the highest average expression level to the lowest. The expression patterns (named 'pattern 1' to 'pattern 3') and the medians of the genes (named 'median 1' to 'median 3') within one cluster are shown in Figure 5. Six Toll-like receptor (TLR) agonists which are C_*p*_G, Pam_2_CSK_4_, Pam_3_CSK_4_, LPS, poly I:C and R848 were used as the treatments, and four gene knock-out mutants and different time points were included to increase the diversity of the TLR-stimulated gene expression data set and the number of measurements [14]. The first four TLR agonists are bacterial-associated, while poly I:C is viral-associated and R848 is anti-viral stimuli. They were used here to stimulate TLR-stimulated macrophages, which represent various pathogen-associated molecular patterns. Among the genes that have been deleted, adapters Myd88 and Ticam1 (product of gene *Myd88 *and *Ticam1*, respectively) could provide a structural platform for the recruitment of kinases and downstream effector molecules, were reported crucial for signaling by most Type I IL-1 receptor(IL1R)/TLR family members [19]. However, Bjökbacka et al. reported that the majority of the host response to LPS is regulated independently of Myd88, and genes appearing to be Ticam1-dependent can be classified as both Myd88-independent and Myd88-dependent [20].

Figure 5 has three main features. First, 'pattern 2' and 'pattern 3' are similar while opposite to 'pattern 1', and 'pattern 2' differs from 'pattern 3' in different average expression level (as shown by the red horizontal bar). Second, there is a plateau in all patterns in the region between points 8 and 9 where either mutant *Myd88*^- ^or *Ticam1*^- ^is used, or no treatment is applied or C_*p*_G is added. These profiles tell us that genes *Myd88 *and *Ticam1 *are crucial for the system (which involves the genes that belong to the three clusters) to response to the external stimuli, and agonist C_*p*_G does not have so much influence on it. Third, whenever LPS or poly I:C is added to the wild type (regions between points 1 and 2, 3 and 4, 5 and 6, 7 and 8, 9 and 10, 11 and 12), there is a sharp drop in 'pattern 1' while there is a peak in 'pattern 2' and 'pattern 3'. This feature indicates that genes from these three groups are sensitive to LPS and poly I:C, and genes that exhibit 'pattern 1' are modulated in an opposite manner as those exhibit the other two patterns. Since poly I:C, LPS and C_*p*_G are TLR-3, TLR-4 and TLR-9 agonists, respectively, and Myd88 and Ticam1 are adaptors involved in TLR-3/4 signaling according to [19], we can deduce that most of the genes belonging to these groups are involved in Myd88-dependent TLR-3/4 signaling cascades.

## Conclusion

This paper presents a novel Beta-Gaussian mixture model, BGMM, for gene clustering from beta distributed and Gaussian distributed data. We developed three types of EM algorithms for BGMM in this study, whose overall performance are similar according to our simulations. We simply chose EM_*h *_as the core of BGMM for further performance test, which was done by comparing BGMM with its two component models, BMM and GMM, with both artificial and real data. Results from artificial data indicate that our joint model works best if the variances of the Gaussian distributed data were not too large, and GO validation of the real case studies show that the joint model yields more comprehensive results no matter what model selection criterion is used and whether the data is carefully chosen or not. For the carefully selected real data, we started from limited known TFs (3 TFs) and ended up with all the TFs (19 TFs) within the tested scope that have the same common features, which demonstrates the usability of one extreme case of BGMM (BMM). After clustering the genes with the 19 TFs, we obtained three distinguished gene groups which might be involved in the Myd88-dependent TLR-3/4 signaling cascades. These results not only tested the performance of the joint model, but also demonstrated its usability in real cases and in some possible applications.

The main contribution of this paper is that it has proposed a framework for multiple data integration through mixture modeling that has not been addressed by anyone else before. The proposed BGMM is designed to integrate beta distributed and Gaussian distributed data. However, the way how those data are incorporated is not limited to the data types that we have used in this study. In principle, data of other parametric distribution can be easily integrated by combining its particular EM algorithm into this framework (given that the optimization method for each case is developed separately). Therefore, the framework proposed in this paper is applicable to many other problems and not limited to the particular problem considered here.

One of the basic assumptions in this paper is that the ground truth clustering for Gaussian and beta distributed data are the same. This is because transcriptional regulation is largely controlled by the TFs that bind to the gene promoters, thus the expression profiles of genes whose regulatory regions are bound by the same/similar factors are expected to be similar. Although the above statement is generally true, it might be violated due to post-transcriptional modifications etc., in which case the method may not be directly applicable. However, if post-transcriptional or other phenomena become a real problem, it can be compensated by integrating more information sources, such as protein-protein interactions, into the proposed clustering framework. On the other hand, if the two data sources do not share the same clustering structure, then an alternative modeling strategy would be needed, such as a hierarchical Bayes model that would model a true clustering structure but allow individual structures for both data types.

Another issue that is worth mentioning is how different data pre-processing and microarray platforms can affect the distribution of gene expression data and, thereby, clustering results. Fortunately, as discussed in section, the alternative distributions we tried on BGMM had a very small effect on the clustering results, suggesting that BGMM is considerably robust to small fluctuations in the distributional assumptions. More importantly, one major advantage of BGMM is its flexibility of easily being extended to other parametric distributions. That is, if in a particular problem data come from different distributions, then one can relatively straightforwardly develop a similar model-based approach as proposed here to model the problem at hand in a precise way by fitting data to those specific distributions.

We employed the diagonal covariance matrix model in the EM algorithm of GMM to reduce the number of parameters to be estimated so that it can be easily applied to large dimensional real data. In particular, the number of parameters in diagonal matrix is *p*_2 _which is remarkably smaller than that of the full covariance matrix . Diagonal covariance matrix automatically assumes no correlations among Gaussian distributed data. So if we want to preserve the correlation information among time series data by the proposed framework without introducing too many new parameters, it is possible, e.g., to develop similar estimation algorithms for a covariance model where off-diagonal constant correlations are assumed or use a more general covariance matrix [5].

In the future, we could improve the proposed model so that it can account for the correlations among gene expressions. We could also integrate more data sources into this framework and apply it to more real problems. In this aspect, we could either combine other data sources into the framework as a component model, or convert them into prior information which can be used to stratify the model [10].

## Authors' contributions

XFD and HL designed the study and developed the methods. XFD implemented the algorithms, did the performance tests, and wrote the manuscript. TE derived the standard EM algorithm for BMM. TE and HL derived the standard EM algorithm for BGMM. XFD, HL, TE and OY-H prepared the manuscript. All authors have read and approved the final manuscript.

## Appendix

### Derivation of *α*'s, *β*'s, and *π*'s

Define



Recall



where , , , ,  and .

### Derivation of *α*'s and *β*'s

Define the parameter vector



Thus the new estimate  is obtained as follows



where  is the Hessian matrix evaluated at .



Note that Ψ and Ψ' represents the digamma and trigamma functions respectively, which are the first and second logarithmic derivatives of the gamma function.

### Derivation of *π*'s


